# A Medical Guide to Nice. Also, Observations on the Climate of Bagneres de Bigorre for Consumptive Patients

**Published:** 1841-07-01

**Authors:** 


I. A Medical Guide to Nice—also, Observations on the Climate of Bagneres de Bigorre, as the most eligible Residence for Consumptive Patients. By *W. Farr*, M.D. 8vo. pp. 174. Churchill, 1841.

II. A Winter in the Azores; and a Summer at the Baths of Furnas. By *Joseph Bullar*, M.D. and *Henry Bullar*, of Lincoln's Inn. 2 Vols. 8vo. with Plates. J. Van Voorst, London, 1841.

III. The Sanative Influence of Climate, &c. By Sir *J. Clark*, Bart. 3d Edition, 1841.

"Men run to and fro, and knowledge is increased." This year we have three treatises on the climates of Naples, Nice, the Azores, and Pyrennees—so that when we take in Madeira, Torquay, Clifton, Undercliff, Hastings, and the Cove of Cork, medical men will be not a little puzzled to determine on the spot which will most effectually prevent or cure phthisis in their patients. Experienced practitioners, and those who have visited the various localities in question, entertain very chastened hopes or expectations from the sanative virtues of climate in phthisical affections; but the afflicted sufferers themselves are always sanguine, and it is necessary therefore, that the physician at home should know as much as possible of the climates abroad that are most resorted to by invalids. We must glance over the publications at the head of this article, and cull as much information from them as we can, for the benefit of our readers.

### I. Nice.

It appears from Dr. Farr, that Nice was resorted to by the Roman invalid. Its climate has not changed for at least one hundred years past—and therefore we may suppose that it is nearly the same as it was two or three thousand years ago. It had once a reputation for the cure of phthisis —but "this reputation, experience has taught us, was not founded in fact."

"There is no doubt that the majority of pulmonary invalids either find no benefit from a residence at Nice, or go away with an augmentation of disease; but it is a difficult task to point out any locality which will be favourable to every consumptive patient." 4.

Dr. Farr still considers Nice a favourable residence for those on whom "this disease steals imperceptibly, as in scrofulous habits."

"For all others, the eastern coast of Spain, from Barcelona to Valencia, and Pau in France, are the best in Europe during the winter, and Bagnères de Bigorre, in the department of the High Pyrenees, in the summer." 4.

Dr. F. thinks that the climate of Nice places the patient under more favourable circumstances for treatment than colder climates—that it keeps up the action of the skin, relieving diseases of the heart and lungs—more especially the former—and that it enables the patient to pass at least six hours daily in the open air, by which he obtains amusement and salutary exercise, incompatible with an English winter. He grieves to say that most people go abroad with two objects in view—health and pleasure. In pursuing the latter, they too often risk the former. "They rush into society with an avidity—frequent crowded and heated rooms—and, after midnight, change a temperature above 80° for one often below 40°. The consequences are, rheumatism or neuralgia among the old—inflammatory affections of some vital organ, perhaps, among the young."

There is considerable variety in the climate of Nice; but the locality under the range of the Cimiez hills, is the warmest part of the environs. "This is the spot for pulmonary invalids, for here they possess all the advantages of an inland climate, and yet, not being the fashionable quarter, i remains unfrequented."

"It was at Cimiez that the Romans fixed themselves, and do we afford proof of superior wisdom in choosing our abodes in more exposed and less salutary situations? Nice has its advantages, and they are unheeded; its disadvantageous positions, and they are resorted to; the result is, that patients leave Nice no better, perhaps worse, than when they arrived, and leave it, terming it a detestable climate." 9.

The author thinks that it would be difficult to point out a spot in Europe more favourable than this for cases of phthisis, "or one where a well directed plan of treatment would have an equal chance of enabling the patient to *throw off the disease*." Alas! we fear that few phthisical patients ever throw off their disease till they have shuffled off the mortal coil along with it. The grand objection urged against Nice is the dryness of the climate. But this dryness, though prejudicial in some diseases, is beneficial in others.

"The easterly wind prevails in the months of March and April: this wind sets in with the first moon in March, called by the natives the blood-red moon; it is severely felt by the invalid and those in delicate health; and even the strong feel and acknowledge its evil tendency. Last season the number of patients of all nations labouring under affections of the chest might have amounted to thirty; the great majority had materially improved their state of health up to this period, and they were daily to be seen, like butterflies in the sun, riding, driving, and walking over hill and dale. I besought those whom I attended, and many that I did not, to quit Nice before the birth of this fatal moon; but they, confident in their amended health and strength, heeded not my counsel, and thought I had overrated the danger; they remained, and the day after this easterly wind began, of the thirty I only met one afterwards, and him I had often previously pronounced to have no disease of the lungs. Were it not for these winds, Nice would be perhaps the finest climate in the world: I do not mean for consumptive cases, but for many other diseases which I shall hereafter mention." 17.

We now come to the diseases benefited by the climate of Nice. Dr. Farr enters into some discussion on the nature of tubercles, and of their nidus. This dissertation we pass over, as almost entirely theoretical. Dr. F. thinks that he has succeeded not only in effecting absorption of tubercles, but in effecting a cure "*even after suppuration had existed for several months*." In the warm and dry climate of Nice, "calomel may be tried with a fair chance of success." He has seen it administered in doses of five grains, night and morning, and continued for six weeks, without producing salivation. The preparation, however, which Dr. F. prefers, is the iodide of iron, which he has exhibited, he says, both at Nice and Bagnères de Bigorre, whose climates are the reverse of one another, with much benefit.

"The largest quantity I have given in a day at Bagnères, is twelve grains; at Nice, eighteen; but in one case, in which it was given in two-grain doses by a native practitioner, it produced, within a fortnight, great emaciation and debility: if, however, it be properly administered, it may be continued for a period of six weeks, or even longer, without any apprehension of dangerous consequences. It reduces the pulse often to the standard of health; the patient gains flesh, health, and strength, under its use; but it should never be given without the advice of some one accustomed to its exhibition: it should not be allowed to accumulate in the system, and, to prevent this, purgatives should be from time to time administered. There is also another preparation of iron which I think I have occasionally used with advantage; this is a chlorate, or the golden drop of the Germans. But, previously to the exhibition of any medicine, either alone or in combination, given with a view to promote the absorption of tubercle, it is highly necessary that the secretions should be properly regulated, and not only that they be good and healthy, but that assimilation should be perfect; in short, that there should be no functional derangement of any organ save the lungs themselves.

Only such cases of phthisis as are in their earliest dawn or invasive stage, when the disease is rather suspected than positively confirmed or established, should be sent here: to the later stages of this malady, the dry, irritating nature of the atmosphere proves prejudicial, and hurries them faster to the tomb." 46.

In cases of scrofula, however, our author gives Nice the preference over all other climates in Europe. In all forms of chronic rheumatism and rheumatic gout, the same locality is eulogised by Dr. Farr.

"I shall briefly notice the other diseases in which benefit may be expected from a residence here: these are—chronic bronchitis, accompanied with expectoration; chronic, and even acute affections of the larynx, those, at least, which have demanded active treatment in England, generally suffer no inconvenience during their sojourn; humid asthma; dyspeptic cases which arise from want of tone in the stomach itself, or from indolent secretion of any organ concerned in the process of digestion; hypochondriacal affections generally; urinary calculi. In hydropic affections I can recommend it strongly: I arrived here last year with so much water in both my legs, that at night I could bury my fingers in them: in less than a month I threw away my bandages, which I had worn for at least three months; and this was entirely the effect of climate, for I employed no remedial means. I have seen several other cases which offered the same result. In secondary syphilis also, whether in the form of eruption or diseased bone, and indeed in eruptive diseases generally, the climate appears to be exceedingly favourable. I succeeded in one case, the brother of a native physician, whose face and nose were covered with what the French call *squamous dartre,* in the short period of twenty-five days; though he had tried every remedy, as suggested in Turin, Paris, and Germany, for a period of two years and a half: two years have now elapsed since his cure, and he still remains well. Diseases of the heart experience great relief; attacks of gout yield very soon to proper treatment; varicose veins; all cases of languid circulation unaccompanied by organic disease; the coughs of the climacteric period also." 57.

In the list of diseases in which the climate of Nice is prejudicial—Dr. F. places all or most cases of phthisis where the complaint is beyond the invasive stage—dry bronchitis—dyspepsia attended with irritation—predisposition to apoplexy or hepatic affections, as also diseases of the kidneys or bladder.

### BAGNERES DE BIGORRE.

This rather uninteresting town, of some seven thousand inhabitants, lies at the foot of the Pyrennees, on the French side, and its brilliant verdure resembles that of England in Summer, contrasting strongly with the burnt-up, dreary, and arid aspect of the Spanish side. The town is rather clean, for a French town, and each street has its murmuring rill running down its centre. The rides and walks about the town are very beautiful.

"Of all the walks, that on the Salut road is the most desirable for the pulmonary invalid; it has a gentle ascent for some distance, is shaded by magnificent poplars, and is well calculated to try his strength, and exercise his lungs." 95.

The great attraction of this place is its peculiar atmosphere, of no great extent, indeed, but sufficient to enable the invalid to take whatever exercise he requires, in every direction except one. The town stands on a bed of argillaceous rock, which being disintegrated by time and winds has the appearance of a wood. This remarkable locality extends, on the south, about a mile and a quarter from the town, and to about an equal distance northward and westward—a space large enough for the usual exercise of a pulmonary invalid. The western parts of the town are best for these patients, being near the Salut road and thermal springs; but for people in health, the Square or Place, is the best.

"In the climate of Bagnères it would appear one can live, as we are accustomed to do in England, upon a mixed diet of animal and vegetable, and can bear very well the strong wines of Spain; the wine found at Bagnères is execrable; I should therefore counsel those who pass through Bordeaux, to secure a provision. The pulmonary patient may even require a small quantity of good claret.

The atmosphere of Bagnères is by no means a desirable one for those in health; one has only to look at the indigenous inhabitants of the soil to be satisfied of this fact: the generality of them, and particularly the working class, are sallow and unhealthy looking, and quite the reverse of handsome. The climate of Bagnères is a decided one; it is anti-irritating and moist, depressing to the healthy, and has a tendency to allay irritation in every organ; and the pulmonary invalid soon finds that this is the kind of atmosphere he ought always to inhale: to him it is decidedly beneficial from the beginning; he escapes what the healthy never fails to experience—the seasoning common to all decided climates, the functions of each organ are more quietly performed, and the organ itself is soon brought into so tranquil a state, that any change in its structure, originating in disease, has a fair chance of being removed by proper remedial means; he is, in fact, placed in the best situation to be treated: climate, however favourable, rarely does more than this. A patient coming to Bagnères with serious disease, and deranged structure of lung, will feel the beneficial influence of the climate while he remains, but, without treatment, he no sooner quits, than he finds his gain is not permanent; his sufferings return, and his disease is just in the state it was on his arrival; he is aware, too late, that he has lost a favourable opportunity, which the advance of disease may not permit a second time to be presented to him." 99.

Dr. F. avers that the invalid subjected to "judicious treatment" goes away "permanently benefited by the *removal of a portion at least, of diseased structure.*" This is a startling sentence, for what pathologist can expect such miracles—except in the hands of the bellows-mender in Hatton Garden ?

The season at Bagnères is short—from the early part of June till the end of September. Then the thermometer falls to 50° in the evenings—and the Pulmonary invalid ought to depart for Pau or Rome. He strongly dissuades such persons from going to Nice. The thermal springs of this place, of considerable reputation in other complaints, have very little in pulmonary affections.

II. The second work on our list is chiefly occupied with the descriptions and remarks of general travellers, and therefore we must endeavour to cull out such portions as bear most on medical matters.

The Azores are comparatively little known to the people of this country, except by the St. Michael oranges, and their enormous onions. St. Michael's was the first foreign soil on which we placed our feet, and we have still a vivid recollection of its fine climate and luxurious vegetation. In the sixth Chapter, our authors take up the Furnas, about twelve miles from Villa Franca, whither they repaired on the 29th day of December. The principal spring or Caldeira, is sulphurous, coming hissing and boiling out of the ground, and diffusing volumes of vapour through the air. It bubbles up through a loose bottom of broken rock, and like the Sprudel, sends up a small column three or four feet in height. The ground beneath trembles, and a pumping sound, like that of a powerful engine, is heard far below. It is evident that the ground is a calcareous crust, like that round the Sprudel, and may, one day, fall in ! At a little distance from the principal Caldeira is a deep smoking pit, at the bottom of which the water is seen boiling furiously. A little way from this is a boiling cauldron of mud, of the "consistency and colour of the creamy sweepings of Regent-street." The vapour that rises from the burning soil is strongly impregnated with sulphuretted hydrogen gas. The iron springs squirt the boiling water through the interstices of volcanic stones, covering them with a thick coating of bright orange rust.

"After looking at the caldeiras, we took our bath, and it certainly was never my good fortune before to bathe in an *invigorating* warm bath. It produced a feeling of strength instead of lassitude, and the skin seemed not alone to have been cleansed, and rendered most agreeably smooth, but to have been actually renewed." 117.

They afterwards took a draught of the iron spa, which sparkles with gas and smacks strongly of steel.

"Most invalids feel that before-breakfast existence is burdensome; but this bath and draught of liquid iron were as a breakfast in producing serenity and happiness, and were more than a breakfast in giving warmth and briskness, and a feeling of health, as of the flowing of younger blood through the veins; and instead of destroying the power of making another they rather increased it many-fold." 118.

The climate of the Furnas in January, is much like that of October in England—pleasant days, but cold mornings and evenings. The climate of Villa Franca on the coast is much milder and more equable.

"As soon as we began to descend the mountain towards the sea, we felt the change of climate, and much enjoyed the delicious warmth of the afternoon sun, whilst riding along the lanes near the coast. But although the climate of this town is nearly unexceptionable, I am not sorry that the English climate is unlike it; for, with the same warmth at home, instead of Englishmen being what they are, they would have grown up a race of lazy, donkey-riding paupers. It is impossible to live here and not to feel the influence of that spirit of laziness which seems to have settled over the island." 131.

Our authors kept a meteorological register of the climate of Villa Franca, during the months of December to April inclusive. In December, the medium heat was $58\frac{1}{2}°$, range 4 degrees.—January 60°, range 3.—Feb. 60°—range 3—March 60°—range 4.—April 59°—range 4.—General average 60°—9′. This shews a fine climate for the foregoing months. It is almost exactly that of the South of England in the month of August.

"The mean temperature of the winter months in St. Michael's, according to these observations, is 2° colder than Madeira; 5° warmer than Lisbon; 13° warmer than Nice; 12° warmer than Rome; and 12° warmer than Naples." 321.

The humidity of St. Michael's is greater than at Madeira or Naples, but less than that of London or Rome. The rain at Villa Franca, on some days, was little more than a mountain scud of a few minutes' duration—at other times it fell heavy. There was only one day of continuous rain during the whole time they were in these islands.

"At the same time, the humidity is so great that your boots grow mouldy in a few days; kid gloves speedily become spotted; books feel damp, and your clothes smell musty.* To prevent these inconveniences, as fires, with one or two exceptions, are nowhere used, except for cooking purposes, the inhabitants are much in the habit of hanging out their clothes in the sun." 323.

This is a considerable drawback on the climate. Our authors observe that this inconvenience might be remedied by lighting a small stove for a couple of hours in the evenings. The skies are rarely cloudless, and the invalid can spend the greater part of the day in the open air.

The natives of St. Michael's are more prone to nervous and atonic, than to inflammatory and sthenic diseases. One of the most prevalent complaints is gastralgia, with little disorder of the digestive functions. This is probably owing to the diet, among the lower classes, which is chiefly cabbages and potatoes, with lard. But these people are also subject to neuralgic affections of other parts of the body. Hypertrophy of the heart is more frequent than in England. Elephantiasis tuberculata is endemic there.

Consumption is extremely rare, though bronchial inflammation is common. It is hardly necessary to say that it is only in the most incipient stages of phthisis, or rather in hereditary predisposition to that malady, that the climate of the Azores can be applicable. But as the climate of Madeira is nearly if not quite as good, and as there are few accommodations for British invalids in the Western Islands, they can hardly be recommended at the present time. The work altogether will be found very amusing to those who visit these beautiful islands.

---

* "I have been informed by a gentleman who spent a winter in Madeira, that the damp in that island produces precisely the same effects."

III. The new edition of Sir James Clark's work is nearly re-written—much having been omitted, in order to make room for new matter. This last is, however, so dovetailed and interwoven with the original matter that it would be extremely difficult to collate the second and third editions, so as to eliminate the additions and corrections made to the present volume. Nor is it necessary. The work is the standard one on climate, and will be in the possession of most intelligent practitioners.

Our readers will remember that Dr. Farr recommended pulmonary invalids to leave Bagnères de Bigorre for Pau or Rome during the Winter. Dr. F. gives us no account of PAU, and therefore we may make a few extracts from Sir James's work, to fill up the hiatus.

PAU is finely situated in the lower Pyrennees upon a ridge of gravelly hills, overlooking an extensive valley to the north. Behind it rise the stately Pyrennees, but with a very gradual ascent, the highest range being 40 miles distant from the town. Sir James is indebted to Dr. Playfair, now of Florence, who resided several years at PAU, for the following:—

"Although the character of the climate of Pau corresponds with that of the South-west of France generally, it possesses some peculiarities which it owes to its topographical situation. Notwithstanding its distance from the coast, it is very much under the influence of the Atlantic. All the changes to which this gives rise extend as far as Pau, though modified, in some degree, by distance, and, still more, by the position of the place with respect to the neighbouring mountains. Calmness, for example, is a striking character of the climate, high winds being of rare occurrence and of short duration.

The mean annual temperature of Pau is 4½° higher than that of London, and about 3° higher than that of Penzance; it is about 5° lower than that of Marseilles, Nice and Rome, and 10° lower than that of Madeira. In *winter*, it is 2° warmer than London, 3° colder than Penzance, 6° colder than Nice and Rome, and 18° colder than Madeira. But in the *spring*, Pau is 6° warmer than London, and 5° warmer than Penzance; only 2½° colder than Marseilles and Rome, and 7° colder than Madeira. The range of temperature between the warmest and coldest months at Pau is 32°: this at London, and likewise at Rome, is 26°; at Penzance it is only 18°, and at Madeira 14°. The daily range of temperature at Pau is 7½°; at Penzance it is 6½°: at Nice, 8½°; at Rome, 11°." 189.

PAU is nearly exempt from the oppressive southerly winds, on the one hand, and the cold north-east winds on the other. Although the climate may be said to be rainy, yet it is not subject to some of the evils which usually attend wet climates. Rain seldom continues more than two days at a time, and is followed in a few hours by warm sunshine—the soil rapidly absorbing the fallen rain. The atmosphere is remarkably free from humidity.

"In October some snow generally falls on the centre chain of the Pyrennees; and, at Pau, this fall is marked by a sudden change of temperature, the weather becoming rainy and chilly. In November the weather clears up, and becomes milder. December and January are cold and dry; frost and slight snow-showers then occur, but the snow does not lie on the ground. The sun is bright and warm; and from twelve till three o'clock, an invalid may generally take exercise. February is milder; but, towards the end of this month the spring rains fall, and the weather is then chilly and disagreeable. March is mild, but variable; though there are no cutting winds. In spring, westerly winds, which are soft and mild, accompanied with rain, alternate with dry easterly winds, also of a mild character. Hence it is, that the vernal exacerbation of inflammatory affections of the stomach and lungs, so commonly observed in other climates, is little felt by invalids at Pau. Vegetation bursts forth in the first week of April, which is a warm month. May resembles April, but is warmer. In June the weather is hot and fine. July, August, and September, are very hot months, the thermometer sometimes rising as high as 94° in the shade; with a very powerful sun, preventing exercise from eight in the morning till seven in the evening." 192.

According to Dr. Playfair, the good qualities of the winter climate of Pau, may be summed up as follows:—

"Calmness, moderate cold, bright sunshine of considerable power, a dry state of atmosphere and of the soil, and rains of short duration. Against these must be placed,—changeableness, the fine weather being as short-lived as the bad; rapid variations of temperature within moderate limits. In autumn and spring there are heavy rains." 193.

PAU is, generally speaking, healthy; rheumatism, being the most prevalent complaint among the natives. Goitre is also common.

"The characteristic quality of the climate, however, is the comparative mildness of its spring, and exemption from cold winds. While the *winter* is 3° colder than the warmest parts of England, and 6° colder than Rome, the *spring* is 5½° warmer than the former, and only 2½° colder than the latter. The mildness of the spring, and its little liability to winds, render this place favourable in chronic affections of the larynx, trachea, and bronchi. In gastritic dyspepsia Dr. Playfair has found it beneficial, and he has seen it useful in a few cases of asthma. With delicate children, also, he found the climate agree well, especially when they removed to the mountains during the summer.

Upon the whole, Pau appears to be the most desirable winter residence in the south-west of France, for invalids labouring under chronic affections of the mucous membranes. In the same class of diseases, the mineral waters of the Pyrennees are also very beneficial; and it may be convenient, and advisable, for the invalid, who has derived benefit from a course of these waters, to pass the winter at Pau, with a view of returning to them in the following season." 193.

PAU is 150 miles from Bourdeaux, and 50 from Bayonne.

